# Iron overload is not the same everywhere: Particularities of iron-metabolism gene mutations in Brazil and a proposal for the investigation and management of iron overload in this population

**DOI:** 10.1016/j.htct.2025.103846

**Published:** 2025-05-15

**Authors:** Paula de Melo Campos, Ana Carolina Toreli, Dulcinéia Martins de Albuquerque, Fernando Ferreira Costa

**Affiliations:** Hemocentro, Universidade Estadual de Campinas - Unicamp, Campinas, São Paulo, Brazil

**Keywords:** Iron overload, Gene mutations, Ethnic groups, Genetic heterogeneity

## Abstract

There is no physiological mechanism for the excretion of iron in humans, and excess iron may lead to severe tissue damage if not adequately treated. Iron overload can be caused by genetic factors (hemochromatosis) or acquired conditions (e.g., ineffective erythropoiesis, transfusions, iatrogenic iron treatment, viral hepatitis, alcohol intake, severe liver disease, metabolic dysfunction), and, in many cases, by a conjunction of these factors. Historically, guidelines for the genetic investigation of patients with iron overload have been based on data obtained from Caucasian individuals in Europe and North America. However, due to the genetic heterogeneity of iron overload gene mutations worldwide, these recommendations might not be applicable to other ethnic groups. This study analyzed previously published genetic data obtained from Brazilian patients with iron overload and found a relevant but small prevalence of *HFE* C282Y/C282Y patients when compared to European populations, while mutations of the *TFR2, SCL40A1, HJV, HAMP, BMP6* and *SLC11A1* genes seem to be important. This study proposes an adapted algorithm for the investigation and management of iron overload in Brazil.

## Introduction

Iron is essential for adequate functioning of the human body. The regulation of its amount in the organism is extremely important, since an excess of iron or iron deficiency has significant adverse effects on the human health. The regulation of the quantity of iron in the human organism is complex and involves the participation of multiple proteins and different biological steps that ultimately lead to the precise control of how much iron should be absorbed in the intestinal tract to keep iron at optimum levels [1,2].

There is no physiological mechanism for the excretion of iron in humans, thus iron that is absorbed in excess or infused during blood transfusion is deposited in different tissues of the body, predominantly in the liver and spleen, and this abnormal deposition of iron, also known as iron overload (IO), leads to severe organ and tissue damage [1, 2, 3]. The multiple mechanisms responsible for causing IO are either hereditary or acquired and, in many cases, not yet completely understood. Possible mechanisms include genetic factors, disorders of red blood cells, transfusion, iatrogenic iron treatment, viral hepatitis, alcohol intake, severe liver disease, metabolic dysfunction (metabolic hyperferritinemia) and possibly a combination of two or more of these factors (Table 1) [1, 2, 3, 4].

**Table 1:** Co-factors associated with iron overload.

Viral hepatitis
Alcohol intake
Fatty liver disease
Hematological disorders (ineffective hematopoiesis)
Insulin resistance Poorly controlled diabetes mellitus Metabolic dysfunction
Exogenous (transfusions, iatrogenic iron reposition)

### Diagnosing iron overload

The first suspicion of IO arises after a simple medical check-up with the observation of elevated serum ferritin levels, almost always the first iron biomarker to be detected. Starting with high serum levels of ferritin, the diagnosis of IO is very frequently not a simple procedure. Ferritin protein is present in a variety of cells, but predominantly in macrophages, with its main function being to store iron in a way that is safe for the cells [5]. The normal concentration of ferritin in serum is very low (30–200 µg/L in females and 30–300 µg/L in males) [5]. Ferritin in plasma is encountered as apoferritin and its function is still not clear. The main problem with the measurement of ferritin is that a number of relatively common clinical conditions may lead to a variable degree of increase in ferritin levels, but without actual IO [3]. These conditions include any kind of inflammatory disorder, diabetes, acute or chronic liver disease, obesity, regular alcohol intake and metabolic syndrome [3]. Thus, the majority of unselected patients with elevated ferritin levels do not have IO. In fact, the first problem regarding the diagnosis of IO is how to identify, among patients with high ferritin levels, those who have IO irrespective of the cause of this condition, thereby differentiating them from those with no IO [6].

The initial diagnostic approach for a patient with a significant increase in ferritin levels verified by at least two measurements (at least 30 days apart) carried out in a reliable laboratory is to estimate transferrin saturation (TS). The presence of hyperferritinemia in the context of normal TS is associated with IO in a very small group of patients [7]. It is important to emphasize that elevated ferritin and concomitantly elevated TS are associated with IO in about 90 % of cases [5]. Thus, it may be assumed that high serum ferritin and high TS (>45 %) is almost always associated with IO. Alternatively, it is possible to assume that high ferritin and normal or reduced TS is not IO, with very rare exceptions. Transferrin is the protein that safely transports iron in blood. TS, indicated as a percentage, can be estimated easily by the ratio between serum iron and total iron-binding capacity. Alternatively, TS can be calculated by the ratio between serum iron concentration and serum transferrin concentration (this ratio should be multiplied by a correction factor of 1.42) [5]. Usually, the normal range for TS in different populations varies from 25% to 45 %. A TS higher than 45 % has been defined as elevated and indicates individuals with probable IO [2,7]. Of note, since the TS is the ratio between serum iron and total iron-binding capacity, expressed as a percentage, TS might be elevated by causes that reduce transferrin levels, such as cirrhosis and dyserythropoiesis, in the absence of IO, which should be take **into** account when analyzing patients [3]. Notably, in patients with hemochromatosis, the elevation of TS occurs significantly earlier than the increase in serum ferritin [8].

Although IO should be considered highly probable, if there are significant increases in both ferritin and TS, an absolute diagnosis of IO can only be made with evidence of higher iron deposits, mainly in the liver [3]. Historically, liver biopsy was used to detect elevated iron deposits, with the advantage of identifying iron distribution in liver cells. However, liver biopsy is a very invasive procedure, and it is no longer routinely carried out for the identification of IO. Currently, the most practical way to estimate liver iron concentration is by T2 magnetic resonance imaging (MRI) [2]. The concomitant findings of elevated serum ferritin levels, TS higher than 45 % and increased liver iron storage estimated by T2 MRI (or, in exceptional cases, liver biopsy) can confirm IO, without doubt, irrespective of the cause.

### Investigation of iron overload

Once IO is identified, the next step is the investigation of the etiology of the iron excess, which is sometimes difficult and complex. In addition, there is some confusion and lack of uniformization in terms of nomenclature and the classification of these disorders. One possible approach to differential diagnoses of IO is presented in Table 2.

**Table 2:** Diagnosis of iron overload (IO) based on the probable etiology.

**Category**	**Condition**
Hereditary	• Hemochromatosis
	• Aceruloplasminemia (low TS)
	• Ferroportin disease (normal or low TS)
	• Hereditary hematological disorders (with or without anemia) with iron overload (ineffective erythropoiesis) – with or without transfusion
	• Iron metabolism gene mutations associated with porphyria cutanea tarda
Acquired	• Acquired hematological disorders (with or without anemia) with iron overload (ineffective erythropoiesis) – with or without transfusion
	• Metabolic hyperferritinemia
	• Excess iron intake, oral or infusion (iatrogenic, chronic dialysis)
	• Severe liver dysfunction
	• Excessive and prolonged alcohol consumption

Before starting an investigation into the abnormalities in the genes involved in the iron metabolism pathway, clinicians should investigate iatrogenic iron intake and previous blood transfusions as possible causes of IO, especially for individuals without a positive family history of IO [9]. In addition, they should identify patients with strong evidence of hematological disease that could lead to ineffective erythropoiesis and increased iron absorption (i.e., hemolytic anemias, myelodysplastic neoplasms, sideroblastic anemias, among others), even if they are not regularly transfused [9]. Given that some rare mutations in heterozygotes for beta thalassemia may show normal red blood cell parameters, in exceptional cases it is recommended to carry out the sequencing of the *β-globin* gene to exclude the association of beta-thalassemia trait and IO with other possible molecular abnormalities related to iron metabolism [10,11]. Porphyria cutanea tarda is also a genetic condition that may be associated with mutations in genes related to iron metabolism and with IO. Although the precise mechanism involved in this association is not completely understood, it is recommended to investigate IO in patients diagnosed with porphyria cutanea tarda [12].

Causes of acquired environmental risk factors for hepcidin deficiency, such as alcohol consumption and end-stage liver disease should also be evaluated [5]. Another possible condition, provisionally called metabolic hyperferritinemia, presents preserved hepcidin production and a total body iron that is generally normal, but in some cases, may present a slight or even moderate IO [13]. Although iron deposits are not very high in metabolic hyperferritinemia, excessive iron may lead to the formation of reactive oxygen species and subclinical inflammation, potentially worsening glucose and lipid metabolism, fibrogenesis and carcinogenesis [13].

In parallel with the evaluation described above, patients should be screened for mutations in the genes of the hepcidin-ferroportin axis, which can lead to a decrease in the production (or activity) of hepcidin [5] or, more rarely, a mutation in the ferroportin gene that results in resistance to its destruction by hepcidin. Recent data using next-generation sequencing for the whole genome to study genetic abnormalities in IO patients have shown that, besides the classic mutations of the *HFE* gene identified as responsible for IO in the majority of patients from Northern Europe and the United Kingdom (the *HFE* mutations), and mutations of *HJV, TFR2, SLC40A1* and *HAMP* genes (the well-known non-HFE mutations), other genes related to iron metabolism are also very probably involved in the pathogenesis of IO [4,14,15], including for example *BMP-6* [16].

Following the suggestion made by a group of experts from the BIOIRON society [8], we recommend the definition of hemochromatosis (HC) as quoted below:

“The term “hemochromatosis” should be reserved for a unique genetic clinical-pathological condition characterized by increased TS, increased serum ferritin, IO in the liver (but not in the spleen), with prevalent involvement of periportal hepatocytes with iron-spared Kupffer cells, and signs and/or symptoms associated with IO. The panelists also emphasized that the term “hemochromatosis” itself implies an IO of genetic origin, which is why they would recommend avoiding the unnecessary use of qualifiers such as “hereditary”, “genetic”, or “primary”. Indeed, genetic defects in the hepcidin/ferroportin regulatory axis (caused by variants in hepcidin regulators, the hepcidin gene itself, or in ferroportin) are responsible for inadequate production or activity of hepcidin or lack of hepcidin responsiveness of ferroportin” [5].

The major problem with the classic classification of HC (Table 3) is its limitation for the inclusion of all the new possible variants that are being described. In addition, the controversial inclusion of ferroportin disease as a subtype of hemochromatosis should also be mentioned.

**Table 3:** Former classification of hemochromatosis.

Classification	Gene involved and location	Inheritance	TS	Other clinical features
Type 1	*HFE* (homeostatic iron regulator)	AR	Increased	Adult-onset; more severe in males; highly variable clinical expression, with predominant liver damage and arthritis
Type 2A	*HJV* (hemojuvelin)	AR	Increased	Earlier onset (e.g., <30 years old); similar severity in both sexes; prevalent cardiac and endocrine involvement
Type 2B	*HAMP* (hepcidin)	AR	Increased	Earlier onset (e.g., <30 years old); similar severity in both sexes; prevalent cardiac and endocrine involvement
Type 3	*TFR2* (transferrin receptor 2)	AR	Increased	Very rare (look for parental consanguinity); clinically similar to Type 1, with an earlier onset
Type 4A	*SLC40A1* (ferroportin)	AD	Low-normal	Adult-onset; IO in the spleen; mild anemia; possible low tolerance to venesection
Type 4B	*SLC40A1* (ferroportin)	AD	Increased	Very rare; in general, clinically similar to Type 1, but more severe/early onset forms are reported

Modified from Girelli et al. [5]

AD: autosomal dominant; AR: autosomal recessive; TS: transferrin saturation.

The new classification proposed by the BIOIRON Society is more flexible and comprehensive, since it allows the inclusion of patients with digenic mutations and HFE/non-HFE compound heterozygosity. Moreover, it allows the inclusion of patients with newly described mutations pending confirmation or not yet identified as causing IO, potentially indicating a provisional diagnosis, as described in Table 4. We strongly recommend the use of this new classification.

**Table 4:** Novel classification of hemochromatosis – from the recommendations of the BIOIRON Society [5].

Novel classification	Molecular pattern	Note
*HFE*-related	C282Y homozygosity or compound heterozygosity of C282Y with other rare *HFE* pathogenic variants or *HFE* deletion	Low penetrance; consider presence of host-related or environmental cofactors for IO In subjects with other HFE genotypes (e.g., C282Y /H63D compound heterozygosity or *p.His63Asp* homozygosity) consider second-line genetic testing for rarer variants
Non-*HFE*-related	Rare pathogenic variants in “non-*HFE*” genes: • *HJV*-related • *HAMP*-related • *TFR2*-related • *SLC40A1* (GOF)-related	Potentially, mutations in any hepcidin-regulatory gene may be causative (the effects of novel mutations should be confirmed through functional and epidemiological studies) Molecular subtypes may be characterized only at specialized centers, but the diagnosis of non-*HFE* related HC is sufficient to start phlebotomies at nonspecialized centers
Digenic	Double heterozygosity and/or double homozygosity/heterozygosity for mutations in two different genes involved in iron metabolism (*HFE* and/or non-*HFE*)	More commonly, C282Y mutation in *HFE* gene might coexist with mutation in other genes; rarely, both mutations involve non-*HFE* genes
Molecularly undefined	Molecular characterization (still) not available after sequencing of known genes (provisional diagnosis)	Patients should be referred (or DNA should be sent) to specialized centers

Since the discovery that mutations of the *HFE* gene could lead to IO [17], the analysis of *HFE* mutations has been the mainstay for the investigation of hereditary IO in the general population. Evidence shows that C282Y homozygosity predisposes to HC, whereas heterozygous C282Y and H63D, and compound C282Y/H63D heterozygosity are reported to be of much less pathological importance if not combined with additional genetic or secondary risk factors [2, 3, 4,14]. Of note, even homozygous C282Y individuals display a heterogeneous clinical presentation, varying from severe HC to a majority of subjects who may never develop symptoms of IO, showing that the natural history of HC relies on individual and environmental variables and not only on the genotype [4]. A recent publication evaluating the clinical penetrance of C282Y/C282Y among 2890 homozygotes from the UK Biobank showed that, by the age of 55 years, only 33.2 % of the men and 21.4 % of the women have a diagnosis of HC [18]. However, several analyses from the UK Biobank suggest that even C282Y/C282Y individuals without a diagnosis of HC may have serious consequences, possibly as a result of IO, even in the absence of clear clinical symptoms [19]. As examples, several recent publications have shown that homozygous C282Y/C282Y men with and without HC demonstrate a 24 % increase in death from any cause, when compared to a control population. In addition, homozygotes have a higher incidence of dementia, a six-fold higher risk of liver fibrosis and cirrhosis and a 10.5-fold higher risk of liver cancer, when compared to a control population [20, 21, 22].

Although the investigation of *HFE* mutations is widely recommended for the screening of IO, the prevalence of *HFE* polymorphisms is highly heterogeneous worldwide. While the C282Y heterozygous mutation is very prevalent in individuals of Northern European ancestry, it is very rare in those from Africa, the Middle East, Asia and Brazilian indigenous population [23]. In a large multiethnic cohort study performed in patients with IO living in Canada or the United States, the Hemochromatosis and IO Screening (HEIRS) study reported a 0.44 % prevalence of C282Y homozygosity in non-Hispanic whites, 0.11 % in Native Americans, 0.027 % in Hispanics, 0.014 % in black individuals, 0.012 % in Pacific Islanders and 0.000039 % in Asians [24].

Brazil is a country of continental dimensions, home to an admixed population. Historically, besides its native indigenous people, Brazil received a great number of immigrants from Western Europe, Africa, Japan and the Middle East [25], leading to regional ethnic particularities and to a very significant genetic heterogeneity. In a pioneer study, carried out in 227 Brazilian individuals from Campinas, the allelic frequency of the C282Y mutation was 1.4 % in the Caucasian population, 1.1 % in the African-derived population, 1.1 % in racially mixed normal controls and 0 % in the original populations (Parakanã Indians) [25]. In another report that included a population of 542 Brazilian healthy blood donors from the city of Sao Paulo, the frequencies of the C282Y and H63D alleles were 2.1 % and 13.6 %, respectively [26], which represent a low incidence, when compared to most European countries [3]. As a comparison, the prevalence of C282Y heterozygosity in European countries increases from the south to north, reaching up to 14.2 % in Ireland [27].

Considering the genetic specificities of the Brazilian population, the investigation of IO in this population is a challenge, since C282Y/C282Y homozygotes seem to represent a small proportion of patients, sharply contrasting with patients from Northern Europe and North America. This study aimed to review available literature related to HC in the Brazilian population and, based on these data, suggest a tentative adaptation of the most recent HC guidelines used worldwide for the specific context of the Brazilian population.

## Method

A literature search of articles was performed in the following databases: PubMed, SciELO, Web of Science, ScienceDirect, Latin American and Caribbean Literature (LILACS) and SCOPUS. The search was independently carried out by two authors (P.M.C. and A.C.T.), using the following descriptors: (“iron” OR “hemochromatosis” OR “HFE” OR “hyperferritinemia”) AND (“Brazil” OR “Brazilian”). The search period was April 2024.

All the articles were completely analyzed. Information regarding the characteristics of the cohort of each manuscript and the incidence of mutations associated with iron physiology was collected.

## Results and discussion

The search resulted in the identification of twelve articles reporting studies that were all performed in Brazilian institutions. The characteristics of the population studied in each manuscript and a summary of results are shown in Table 5. The prevalence of *HFE* mutations in healthy Brazilian subjects varied from 1.1–3.3 % for C282Y mutations, and from 7.5–13.6 % for H63D. The only study that evaluated the prevalence of *HFE* mutations in an indigenous population from Brazil, found no mutations of the *HFE* gene [25]. Of Brazilian subjects with definitive evidence of IO (increased ferritin and TS), the prevalences of *HFE* mutations were 13–53 % and 0–15 % for the C282Y/C282Y and C282Y/H63D genotypes, respectively. Only three studies evaluated additional mutations related to iron metabolism: (a) Bittencourt et al. [28] found no mutations of the *TFR2* and *SCL40A1* genes in a cohort of 19 patients; (b) Santos et al. [29] described prevalences of mutations of the *TFR2* (7.8 %), *HJV* (5.8 %), HAMP (1.9 %), and *SLC40A1* (1.9 %) genes in 51 patients with IO; (c) Toreli et al. [30] analyzed 20 genes involved in iron physiology from whole exome results, and found mutations in the following genes: *TFR2* (5.0 %), *HAMP* (2.5 %), *BMP6* (12.5 %), and *SLC11A1* (10.0 %). Although the numbers of patients studied in all of these reports were small, the results strongly indicate that the percentage of patients in the Brazilian population with IO who are C282Y/C282Y homozygotes is probably <20 %. The studies also indicate that other genetic alterations (non-*HFE*), either in isolation or in combination with *HFE* mutations, may be important in the Brazilian population. Table 6 shows the results of the whole exome sequencing of 20 genes involved in iron metabolism observed in 40 patients with IO in Brazil [30]. In this cohort, C282Y/C282Y homozygotes represent only 13 % of the patients, whereas other mutations (some described for the first time) in genes related to iron metabolism were found, including the *BMP6, TRF2*, and *HAMP* genes [30].

**Table 5:** Prevalence of mutations in iron overload-related genes in different Brazilian populations.

Study	Population	Method	*HFE* C282Y	*HFE* H63D	*HFE* C282Y/H63D	*TFR2*	Additional mutations
Agostinho, M. F. [25]	Healthy volunteers: *n* = 227	PCR-RFLP	1.4 % White 1.1 % blacks 1.1 % racially mixed 0 % Amerindians	16.3 % White 7.5 % blacks 9.8 % racially mixed 0 % Amerindians	NP	NP	NP
Bittencourt P. L. [31]	Patients with IO: *n* = 15	PCR-RFLP	53 % *n* = 8 (282/282) 7 % (*n* = 1) (282/WT)	7 %, *n* = 1	0 %	NP	NP
Barbosa, K. V. B. D. [32]	Blood donors with IO: *n* = 10 (screened from 1039 healthy blood donors)	PCR-RFLP	10 % *n* = 1 (282/282)	20 % (63/WT), *n* = 2 10 % (63/63), *n* = 1	0 %	NP	NP
Cançado R. D. [33]	Patients with IO: *n* = 35	PCR-RFLP	14 % (282/282) 17 % (282/WT)	29 % (63/WT) 3 % (63/63)	11 %	NP	NP
Terada, C. T. [34]	Blood donors: *n* = 108	PCR-RFLP	2.2 %	NP	NP	NP	NP
Bittencourt P. L. [28]	Patients with IO: *n* = 19	Haemochromatosis StripAssay A	47 % (282/282)	11 %	5 %	0 %^a^	*SCL40A1*^a^: 0 %
Santos, P. C. et al. [26]	Blood donors: *n* = 542	PCR-RFLP	2.1 %	13.6 %	0.7 %	0 %^b^	NP
Santos, P. C. et al. [29]	Patients with IO: *n* = 51	Bidirectional DNA sequencing of HFE, *HJV, HAMP, TFR2* and *SLC40A1*	21.6 % (282/282) 7.8 % (282/WT)	21.6 % (63/WT) 3.8 % (63/63)	11.7 %	7.8 %	*HJV*: 5.8 % (*n* = 3) *HAMP*: 1.9 % (*n* = 1) *SLC40A1*: 1.9 % (*n* = 1)
Leão, G. D. R. [35]	Patients with hyperferritinemia: *n* = 299	PCR-RFLP	2.67 % (282/282) 4.35 % (282/WT)	31.44 % (63/WT) 8.03 % (63/63)	5.02 %	NP	NP
Alves, L. N. R. [36]	(a) Healthy volunteers: *n* = 120 (b) Patients with IO: *n* = 20	PCR-RFLP	(a) 0 % (282/282) 3.33 % (282/WT) (b) 5 % (282/282) 25 % (282/WT)	(a) 20.83 % (63/WT) 0.83 % (63/63) (b) 5 % (63/WT) 5 % (63/63)	(a) 0 % (b) 15 %	NP	NP
Kersting, N. [37]	Patients with hyperferritinemia: *n* = 214	PCR-RFLP	14.0 % (282/282)	7.9 % (63/63) 21.5 % (63/?)	11.8 %	NP	NP
Toreli et al. [30]	Patients with IO: *n* = 40	Exome sequencing of 20 genes implicated in iron physiology	13 % (282/282) 17.5 % (282/WT)	50 % (63/WT)	13 %	5 % (*n* = 2)	*HAMP*: 2.5 % (*n* = 1) *BMP6*: 12.5 % (*n* = 5) *SLC11A1*: 10 % (*n* = 4)

PCR-RFLP, polymerase chain reaction - restriction fragment length polymorphism analysis; IO, iron overload; WT, wildtype; NP, not performed.

a Only TfR2 E60X, M172K, Y250X, AVAQ594–597del, and SCL40A1 N144H and V162del mutations were analyzed.

b Only TFR2 Y250X (*n* = 212) and TFR2 Q690P (*n* = 516) were analyzed.

**Table 6:** Iron overload in Brazil.

Patient	Gender	Gene	Mutation		Comorbidity
1	M	*TfR2* *HFE* *HBB*	p.Arg752His p.His63Asp p.Gln40	HET HET HET	Thalassemic trait
2	M	*BMP6* *HBB*	p.Pro95Ser p.Gln40	HET HET	Thalassemic trait
3	M	*CYBRD1* *HEPH*	p.Arg226HIS p.Ala649Thr	HET HOM	
4	M	*HFE*	p.Cys282Tyr	HOM	
5	M	*HFE* *HFE* *SLC11A1*	p.Cys282Tyr p.His63Asp p.Pro234Arg	HET HET HET	
6	M	*HAMP*	c.−72C>*T*	HET	
7	M	*HFE*	p.Cys282Tyr	HET	
8	M	*HFE* *HFE*	p.His63Asp p.Cys282Tyr	HET HET	
9	M	–	–	–	
10	M	*SLC11A1* *HFE*	p.Arg397Cys p.His63Asp	HET HET	
11	F	*BMP6*	p.Arg257His	HET	Hepatic cirrhosis
12	M	*HEPH* *HFE*	p.Ala649Thr p.His63Asp	HOM HET	
13	M	*HFE*	p.Cys282Tyr	HOM	
14	M	–	–	–	Hepatic cirrhosis
15	M	*HFE*	p.Cys282Tyr	HET	
16	F	*HFE* *HFE* *UROD*	p.Cys282Tyr p.His63Asp p.Pro62Leu	HET HET HET	Porphyria Cutanea Tarda (PCT)
17	M	*HAMP* *HFE*	c.−72C>*T* p.His63Asp	HET HET	
18	M	*HFE*	p.His63Asp	HET	
19	M	*HFE*	p.His63Asp	HET	
20	M	*TFR2* *HFE*	p.Arg752His p.His63Asp	HET HET	HIV; Hepatitis C; PCT; Hepatic cirrhosis
21	M	*HFE*	p.His63Asp	HET	Alcohol abuse
22	M	–	–	–	
23	M	*HFE*	p.His63Asp	HET	
24	M	–	–	–	
25	M	*HFE*	p.Cys282Tyr	HET	
26	M	*HFE*	p.His63Asp	HET	Hepatitis C
27	M	*HFE*	p.Cys282Tyr	HOM	
28	M	*HFE*	p.His63Asp	HET	
29	M	*AHSP*	p.Asn75Ile	HET	
30	M	*BMP6* *HFE* *HFE*	p.Arg257His p.Cys282Tyr p.His63Asp	HET HET HET	
31	M	*FTH1* *HFE*	p.Lys54Arg p.His63Asp	HET HOM	
32	M	*TFR2* *HFE*	p.Glu491Glu p.His63Asp	HET	
33	M	*BMP6*	p.Val394Met	HET	
34	M	*SCL11A1* *HFE*	p.Pro231Leu p.His63Asp	HET HET	
35	F	*HFE*	p.His63Asp	HOM	
36	M	*BMP6* *HFE* *HFE*	p.Leu71Val p.Cys282Tyr p.His63Asp	HET HET HET	
37	M	*HFE* *SLC11A1* *SLC11A1*	p.Cys282Tyr p.Ala244Thr p.Ala244Val	HOM HET HET	
38	F	*HFE*	p.His63Asp	HET	PCT
39	M	*HFE*	p.Cys282Tyr	HOM	
40	M	*TF* *HFE* *UROD*	p.Arg343Trp p.His63Asp p.Pro62Leu	HET HET HET	PCT

M, male; F, female; HET, heterozygous mutation; HOM, homozygous mutation; HIV, human immunodeficiency virus.

Genes - *TfR2* (transferrin receptor 2), *HFE* (homeostatic iron regulator), *HBB* (hemoglobin subunit beta), *BMP6* (bone morphogenetic protein 6), *CYBRD1* (cytochrome b reductase 1), *HEPH* (hephaestin), *SLC11A1* (solute carrier family 11 member 1), *HAMP* (hepcidin antimicrobial peptide), *UROD* (uroporphyrinogen decarboxylase), *AHSP* (alpha hemoglobin stabilizing protein), *FTH1* (ferritin heavy chain 1), *TF* (transferrin).

Toreli et al. [30].

**Figure 1:**
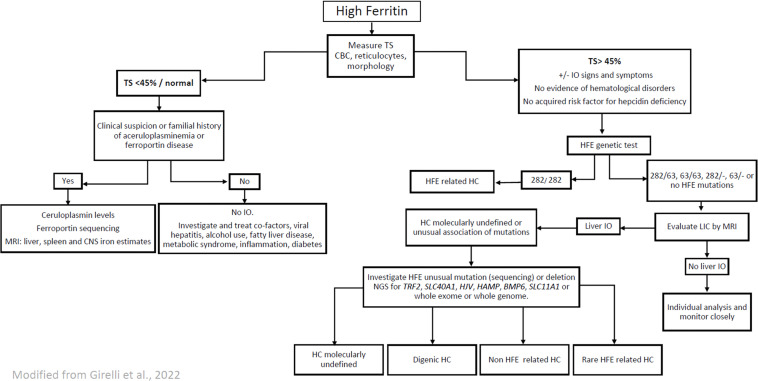
Algorithm proposed for the diagnosis of haemochromatosis in Brazilian patients. TS, transferrin saturation; CBC, complete blood count; IO, iron overload; HC, hemochromatosis; LIC, liver iron concentration; MRI, magnetic resonance imaging; CNS, central nervous system Genes, *HFE*, homeostatic iron regulator; *TfR2*, transferrin receptor 2; *SLC11A1*, solute carrier family 11 member 1; *HJV*, hemojuvelin BMP, co-receptor; *HAMP*, hepcidin antimicrobial peptide; *TFRC*, transferrin receptor; *BMP6*, bone morphogenetic protein 6; *BMP2*, bone morphogenetic protein 2; *SLC40A1*, solute carrier family 40 member.

Taking into account the genetic characteristics of the Brazilian population described above, some modifications to the 2022 European Association for the Study of the Liver (EASL) Clinical Practice Guidelines on Haemochromatosis[3] can be suggested in order to adapt the guidelines to the Brazilian context, as follows (Figure 1).

### Proposal of a new algorithm for the investigation and management of iron overload in the Brazilian population

#### Who should be screened for iron overload?

The suspicion of IO is usually raised by the finding of elevated ferritin levels, defined as >300 mg/L in males and >200 mg/L in females [38]. However, hyperferritinemia may potentially be found in 5.9–19.0 % of healthy individuals [24]. Moreover, since ferritin is an acute-phase reactant protein and is released in the presence of inflammation and from necrotic or lysed cells, it is common to observe its elevation in individuals who do not have IO [3]. Therefore, it is essential to associate ferritin results with transferrin saturation (TS). A TS >45 % in men and women has been defined as elevated [3] indicating that individuals should be investigated for IO. Family members of patients diagnosed with HC and patients with increased liver iron, evident by liver biopsy or by MRI, should undergo biochemical and genetic testing [3,4].

Patients with high ferritin levels but with TS <45 % very probably do not have IO and, as already mentioned, other factors should be investigated to determine the cause of hyperferritinemia. Patients with high ferritin levels and with TS <45 % without a clear cause, should be observed at least annually with measurements of serum ferritin and TS.

An elevated serum ferritin in association with low TS may occur in three pathological states that might result in iron tissue deposition: metabolic hyperferritinemia, ferroportin disease and aceruloplasminemia [39]. Metabolic hyperferritinemia should be suspected when there is an insulin resistance syndrome with mild hepatic iron excess. Ferroportin disease (A-form) is characterized by predominant macrophage iron excess and absent or mild iron-related complications. Hereditary aceruloplasminemia is associated with major hepatocyte IO and diabetes mellitus, with the common finding of anemia and the presence of a neurological syndrome [39]. Once there is a suspicion of one of these conditions, targeted clinical and/or genetic investigation is mandatory, in conjunction with the investigation of tissue iron deposition by T2* MRI [3].

#### Identifying risk factors and comorbidities

Ferritin levels are often increased in chronic inflammatory conditions, liver disease, high alcohol consumption, obesity fatty liver, insulin resistance, poorly controlled diabetes mellitus*,* and metabolic dysfunction [13,40]. Unlike patients with HC, patients with metabolic hyperferritinemia have preserved production of hepcidin, and total iron body stores range from normal to a moderate level of IO [13]. Although iron levels in metabolic hyperferritinemia are not as high as those seen in HC, excess iron leads to the formation of reactive oxygen species and subclinical inflammation, worsening glucose and lipid metabolism, fibrogenesis and carcinogenesis [13].

In addition to the metabolic causes described above, active liver disease should also be evaluated in patients with evidence of IO. Alcoholic liver disease and hepatitis C infection may lead to IO and reticuloendothelial iron deposition [39]. Of note, iron removal by phlebotomy has been reported to improve the rate of response to interferon treatment in hepatitis C patients [41], although with the use of direct-acting antivirals, IO seems not to be a barrier to achieve response [42].

Finally, in patients diagnosed with IO, hematological diseases that lead to ineffective erythropoiesis and increased iron absorption (i.e., hemolytic anemias, myelodysplastic neoplasms) should be regularly investigated, even if they are not regularly transfused.

#### Genetic investigation of iron overload in Brazil

The classic *HFE* mutations, C282Y and H63D, should, of course, be investigated in patients suspected of IO, since *HFE* C282Y homozygosity can result in a potentially severe phenotype of HC, and the heterozygous phenotypes of C282Y/–, H63D/– and C282Y/H63D may also result in IO when combined with other genetic and environmental risk factors [2, 3, 4,14]. It is important to emphasize that the association of C282Y/H63D should not be classified as classic *HFE* hemochromatosis since this genotype has minimal or no clinical penetrance. In patients with this association (C282Y/H63D), mutations and deletions in *HFE* and non-*HFE* genes should be investigated. Furthermore, causes of liver disease should be investigated. Moreover, since the incidence of *HFE* mutations in Brazilian patients with HC is much lower than that observed in Northern European populations, investigation of other mutations in genes involved in iron metabolism should be considered for patients that do not harbor *HFE* mutations or who are not C282Y homozygotes. Taking into account previous Brazilian studies that evaluated non-*HFE* mutations in IO patients [28, 29, 30], the investigation of mutations of the *TFR2, SCL40A1, HJV, HAMP, BMP6* and *SLC11A1* genes might be valuable. However, it is very important to emphasize that, in the few available studies in Brazil, the number of identified patients with non-*HFE* mutations is quite low and in many patients with IO, no mutations were found. This strongly indicates that further studies in larger multicentric cohorts should be carried out to fully clarify the mutation spectrum of IO in Brazil. If available, a broader study with next-generation sequencing, by whole exome or complete genome sequencing, should be carried out.

If a diagnosis of early-onset HC is suspected (within the second or third decades of life, family history, hypogonadotropic hypogonadism or unexplained heart failure), sequencing of the *HAMP* and *HJV* genes is recommended [39]. If there is clinical suspicion of hereditary aceruloplasminemia, ceruloplasmin levels should be evaluated [39].

As already mentioned, in some cases, the genes for porphyria cutanea tarda and the *β-globin* gene (or *HBB* gene) should be sequenced when clinically suspected.

#### Management of iron overload

If not adequately treated in an early phase, excess free iron may lead to progressive tissue damage, with subsequent cardiac failure, cirrhosis and endocrine disfunction [1]. Additionally, as already described, high serum levels of ferritin have been associated with the amount of hepatic lipid accumulation, the severity of insulin resistance and features of metabolic dysfunction [13]. For this reason, decisions require individualized clinical assessment and patients with evidence of IO should be treated appropriately even if a specific genetic mutation cannot be identified. Ideally, patients with suspected IO (high ferritin and high TS) should be evaluated for liver iron concentration at diagnosis using T2* MRI in order to estimate the amount of iron deposition and to guide treatment [2]. Patients diagnosed with early-onset forms of HC should also be evaluated for cardiac iron deposition by cardiac T2* MRI [3].

Patients with no (or minimal) symptoms, but with persistently increased levels of serum ferritin, TS higher than 45 % and increased liver iron estimated by MRI, should be treated by phlebotomy in order to prevent organ damage. The management of patients is determined by their phenotypic presentation and the presence of associated cofactors, not by the genotype alone [2,3,13]. Treatment of risk factors and comorbidities is mandatory [2]. Alcohol intake should be restricted [3]. The initiation and maintenance of a schedule of phlebotomies is determined by serum iron studies (ferritin and TS), and the MRI, if available [2].

During the initial phase, the performance of phlebotomies in the range of 400–500 mL, according to body weight, weekly or every two weeks has been proposed [38]. The main treatment goal is to lower ferritin levels, with a serum ferritin target of 50 µg/L, although, in real-life, levels up to 100 µg/L are acceptable, even in the induction phase. During the maintenance phase, one phlebotomy is performed every 1–4 months, depending on the patient’s iron status [38], with the target serum ferritin being around 50–100 µg/L^3^. Although the reduction of TS to <50 % should be considered as highly desirable, in some patients this reduction is slow and difficult to achieve. Thus, the real target to guide treatment must be ferritin levels and not TS. The clinical implications of the maintenance of high TS for long periods with concomitantly low ferritin levels are not yet well understood [43,44]. If hemoglobin concentrations are <12 g/dL, the volume or frequency of phlebotomy should be decreased; if hemoglobin falls below 11 g/dL, phlebotomy should be discontinued until the causes of anemia can be assessed and adequately treated [3].

The role of iron depletion by phlebotomy in metabolically induced high ferritin levels remains an open question. In these patients, the maintenance of ferritin levels <50 µg/L does not seem to ameliorate metabolic endpoints (i.e., glucose control), nor transaminase levels or the liver fibrosis score [38]. However, if there is evidence of moderate or severe iron accumulation (defined by ferritin >1,000 ng/mL, or metabolic hyperferritinemia with a MRI estimative of >74 µmol/g of iron in the liver), phlebotomy should be considered, together with close monitoring of hemoglobin levels and adverse events [13] (using a cautious protocol of phlebotomy).

Since iron-chelating agents are not superior to therapeutic phlebotomy and usually cause adverse events, they are not routinely recommended for the treatment of IO in patients who are not anemic and who do not have other difficult issues regarding phlebotomy (inaccessible veins, needle phobia) [2,3]. However, iron-chelating agents can be used in association with phlebotomies in patients with severe IO, such as in cases of early-onset HC and cardiac iron deposition [3,38]. Proton pump inhibitors can also be an adjuvant to phlebotomy for some patients, since they reduce intestinal iron absorption [3]. Apparently, dietary heme or nonheme iron restrictions have no significant relationships with iron body content, and there is no strong evidence that dietary restrictions have an important role in IO treatment [45]. However, if the patient accepts the suggestion, black tea with meals and a vegetarian diet may be prescribed since these seem to be beneficial for some patients [46,47]. Erythrocytapheresis is very effective and can be considered where available to treat HC patients, however it is more expensive and less available than phlebotomy [38].

#### Special considerations regarding the Brazilian economic scenario

When guidelines are proposed, they must consider the best available evidence to help clinicians in taking the most appropriate decisions in specific clinical contexts. In the scenario of IO, advanced tools are certainly helpful for the diagnosis and management of this condition, particularly the complete genetic identification of mutations and quantification of tissue iron deposition by T2* MRI.

A frequent problem that many physicians may encounter in a country like Brazil, with limited resources and unequal access to technology in public health services is the impossibility to obtain an MRI estimate of liver iron or access to molecular diagnosis. Given the harmful consequences of untreated IO, as strongly suggested by published reports [43,44], it is recommended that patients with a very probable IO (elevated serum ferritin levels (>400 µg/L in men and >300 µg/L in women) for at least six months and TS higher than 45 %) should be treated with therapeutic phlebotomy targeting a serum ferritin of 50–100 µg/L, even if genetic investigation or MRI cannot be performed. We recognize that the numbers indicated here are somewhat arbitrary, but they are based on available evidence in the literature and also on the personal experience of several experts in the field.

The patients classified with "very probable IO" should be divided into two groups, the first with levels of ferritin higher than 1,000 µg/L and the second with ferritin between 400 µg/L and 1,000 µg/L. For those with ferritin higher than 1,000 µg/L and of course, high TS, there are a number of indications that liver damage secondary to IO may be very probable, and phlebotomy should be carried out following the traditional protocol, as described earlier. On the other hand, for patients who have ferritin levels of between 400 µg/L and 1,000 µg/L and elevated TS, the evidence supporting phlebotomies is weaker and these individuals should undergo careful individual analysis regarding their clinical status. However, a recent relevant study showed that patients with classic C282Y/C282Y *HFE*-related hemochromatosis with ferritin levels of between 300 µg/L and 1,000 µg/L demonstrated a significant clinical improvement when submitted to repeat phlebotomies. Thus, for individuals with intermediately elevated ferritin levels (between 400 µg/L and 1,000 µg/L), a specialized and carefully designed treatment protocol is proposed. Phlebotomies should be undertaken every three or four weeks. Hemoglobin levels must be monitored before each procedure and ferritin measured every 30 days. The treatment target should be ferritin of 100 µg/L. If Hb is ≤12 g/dL, the procedure should be stopped. After three or four phlebotomies the patient should be carefully reevaluated. After the target of 100 µg/L is reached, patients should be kept in the usual maintenance phase, with 2–4 phlebotomies per year, and checked for ferritin and TS every six months.

## Conclusion

IO is a state in which an excess of free plasma iron leads to progressive cellular and tissue damage. The clinical phenotype relates to a conjunction of genetic mutations and co-factors, including comorbidities and environmental factors. The prevalence of mutations in iron physiology-related genes has a very important variation in different populations. In Brazil, the incidence of *HFE* mutations is much lower than that observed in the Northern European and North-American populations. Based on several reports in the literature, it is recommended that the genetic investigation of IO in Brazil be extended, and the investigation of mutations of the *TFR2, SCL40A1, HJV, HAMP, BMP6* and *SLC11A1* genes should be considered for individuals who are not homozygous for the C282Y mutation. It should be underscored that these proposals are still based on a limited sample population and future studies conducted in larger cohorts are important to strengthen these recommendations. The treatment decision requires an individualized clinical assessment and patients with evidence of IO should be treated appropriately, even if a specific genetic mutation cannot be investigated or identified.

## Authors contribution

PMC performed the references selection, elaborated the tables and the figure, and wrote the manuscript. ACT performed the references selection, collected the genetic data from the references and reviewed the manuscript; DMA reviewed all the genetic data from the references, edited the tables and the figure and reviewed the manuscript; FFC conceived the format of the manuscript and wrote the text.

## Conflicts of interest

The authors declare no conflicts of interest.
